# Health-related quality of life and related characteristics of persons with spinal cord injury in Nigeria

**Published:** 2019-04-04

**Authors:** Munir Abdullahi Nasidi, Mukadas O. Akindele, Aminu A. Ibrahim, Aisha Ahmad Ahmad, Aliyu Musa

**Affiliations:** Department of Physiotherapy, Faculty of Allied Health Sciences, College of Health Sciences, Bayero University, Kano, Kano State, Nigeria

**Keywords:** Health-Related Quality of Life, Nigeria, Spinal Cord Injury

## Abstract

**Background:** Spinal cord injury (SCI) is impairment of the spinal cord resulting in numerous health problems that considerably affect the quality of life (QOL) of the patients. Moreover, a number of sociodemographic and clinical characteristics may influence the persons’ health-related quality of life (HRQOL). However, there is limited information on the HRQOL and related characteristics among affected persons living in Nigeria. This study explores the HRQOL and related characteristics of persons with SCI in Kano, Northwestern Nigeria.

**Methods:** A prospective cross-sectional survey of 41 subjects with SCI and 40 age and gender matched healthy subjects was conducted from January to December 2016. Subjects' sociodemographic and clinical characteristics and HRQOL (using the SF-36 questionnaire) were collected and analyzed.

**Results:** The majority of the subjects were men in both the SCI (85.4%) and healthy (82.5%) groups. The mean injury duration was 28.4 ± 20.2 months. Road traffic accident (46.3%) was the leading cause of injury with paraplegia (70.7%) being the most frequent level of injury. A greater number of the subjects (43.9%) had a complete impairment. Subjects with SCI had significantly lower HRQOL in the domains of general health, physical functioning, bodily pain, social functioning, role-emotional, and mental health compared to healthy controls. Gender, level of injury, and severity of injury were commonly found to be related to lower HRQOL scores.

**Conclusion:** Persons with SCI from Kano, Northwestern Nigeria have lower HRQOL across various domains compared to healthy controls. Common factors related to lower HRQOL scores were gender, level of injury, and severity of injury. There is a need for optimal rehabilitation for persons with SCI in Kano, Northwestern Nigeria.

## Introduction

Spinal cord injury (SCI) is impairment in the spinal cord resulting in numerous health problems that affect the physical and psychosocial well-being of the patients.^1^ It does not only affect the health of the individual, but also imposes a considerable economic burden on families and society as a whole.^2^

Moreover, it mainly affects the young age groups of the population who are vital to national economy.^3^ Since SCI typically leads to chronic disability and increased risk of secondary health complications that change the lifestyle of the individual,^4^ optimal healthcare is an impetus to improve the health status of the affected individuals.

The effects of SCI on the affected persons can be assessed through physiological, psychological, social functioning and participation after discharge from the hospital.^5^ This could enable healthcare professionals to render rehabilitation interventions towards functional performance, total well-being, and quality of life (QOL). SCI is usually associated with health complications and problems that negatively affect the QOL of the patients. Information on the impact of SCI on QOL is valuable as it enables suitable monitoring and organization of a healthcare delivery system.^6^

A number of studies have shown that persons with SCI experience lower levels of health-related quality of life (HRQOL) when compared with normative data and healthy populations.^7^^-^^11^ Moreover, several sociodemographic and clinical characteristics have been linked with lower scores of HRQOL among persons with SCI.^7^^,^^9^^,^^10^^,^^12^^-^^15^ Furthermore, these factors may vary in different countries due to cultural, social, economic, and environmental characteristics. 

It is worth noting that the majority of studies on the HRQOL among persons with SCI were carried out in developed countries.^7^^,^^9^^,^^10^^,^^12^ However, there has been a limited number of research in this area in developing countries, particularly African nations like Nigeria. The purpose of this study was to explore the HRQOL and related characteristics of persons with SCI in Northwestern Nigeria.

## Materials and Methods

This study was a cross-sectional prospective survey of all patients with SCI in Kano State, Northwestern Nigeria, from January to December 2016. Ethical approval was obtained from the ethical committees of Aminu Kano Teaching Hospital, National Orthopaedic Hospital Dala, and the hospital management board of Kano State. Written informed consent was obtained from all the subjects after receiving written and verbal information about the study. 

The participants were persons with SCI, at least 6 months post-injury and post-discharge, who were referred to physiotherapy outpatient units of Aminu Kano Teaching Hospital, National Orthopaedic Hospital Dala, Murtala Muhammad Specialist Hospital, and Muhammad Abdullahi Wase Teaching Hospital in Kano State. They were recruited using purposive and snowball sampling techniques. The exclusion criteria consisted of less than 18 years of age, head and SCI, mental problems and physical disability prior to SCI, and a history of alcoholism/addiction to illegal drugs.

Healthy (control) subjects matched in age and gender were recruited from the neighboring hospital communities in Kano State through flyers pasted in various locations and by general word of mouth. They were excluded if their age was below 18 and had an observable physical disability or serious medical illness.

***Sociodemographic and clinical characteristics: ***Sociodemographic (age, gender, marital status, educational level, and occupational status) and clinical (duration of injury, causes of injury, level of injury, severity of injury, and presence of pressure sore) characteristics were obtained directly from the subjects during assessment and were recorded in pre-prepared forms. The American Spinal Injury Association (ASIA) Impairment Scale^16^ was used to evaluate the severity of injury using the following classifications: ASIA-A (complete injury): no preserved sensory or motor function below the neurological level; ASIA-B (incomplete injury): only sensory function is preserved below the neurological level and includes the sacral segments S4–S5; ASIA-C (incomplete): preserved motor function in which more than half of key muscles below the neurological level have a muscle grade < 3; ASIA-D (incomplete): preserved motor function in which at least half of the key muscles below the neurological level have a muscle grade of 3 or more; and ASIA-E (normal): sensory and motor function are normal.^16^

***HRQOL:*** Data on HRQOL was collected using the SF-36 health questionnaire.^17^ The SF-36 is widely used for evaluating the health status of individuals with SCI. It contains the 8 health domains of general health (GH), physical functioning (PF), role-physical (RF), bodily pain (BP), vitality (VT), social functioning (SF), role-emotional (RE), and mental health (MH). These domains provide two component summary scores, physical component summary (PCS) and mental component summary (MCS). The PCS comprises domains of GH, PF, RP, and BP, while the MCS comprises domains of VT, SF, RE, and MH. The total score range of the SF-36 is 0-100 with higher scores signifying better HRQOL.^17^

Descriptive analysis was used to describe the data using frequency and percentage for categorical variables, and mean and standard deviation for continuous variables. Comparison between subjects with SCI and healthy controls was carried out using the Mann-Whitney U test and chi-square test for continuous and categorical variables, respectively. Spearman’s rank correlation was used to investigate relationships between continuous variables. Kruskal-Wallis and Mann-Whitney U tests were applied to investigate relationships between continuous and categorical variables where applicable. Domains of HRQOL and PCS and MCS scores were the dependent variables, and related characteristics (age, gender, marital status, educational level, occupational status, duration of injury, level of injury, and severity of injury) were the independent variables. All statistical analyses were conducted in SPSS for Windows (version 21, IBM Corporation, Armonk, NY, USA) with a significant level of P < 0.05.

## Results

***Sociodemographic and clinical characteristics: ***Sociodemographic and clinical characteristics of the study groups are presented in table 1. There was no statistically significant difference between the subjects with SCI and healthy subjects in terms of age, gender, marital status, educational level, and occupational status (P > 0.05). The majority of subjects were men in both the SCI (85.4%) and healthy (82.5%) groups. The mean injury duration was 28.4 ± 20.2 months. Road traffic accident (46.3%) was the leading cause of injury with paraplegia (70.7%) being the most frequent level of injury. The majority of subjects with SCI (43.9%) had ASIA-A, and more than half of them developed pressure sores (56.1%).

**Table 1:** Sociodemographic and clinical characteristics

**Variable**	**SCI group (n = 41)**	**Healthy group (n = 40)**	**P**
Age (years) (mean ± SD)	37.0 ± 11.7	35.7 ± 11.5	0.545
Duration of injury (month) (mean ± SD)	28.4 ± 20.2	NA	
Gender [n (%)]			0.480
Male	35 (85.4)	33 (82.5)	
Female	6 (14.6)	7 (17.5)	
Marital status [n (%)]			0.564
Married	28 (68.3)	27 (67.5)	
Single	13 (31.7)	13 (32.5)	
Educational level [n (%)]			0.507
Formal education	21 (51.2)	23 (57.5)	
Non-formal education	20 (48.8)	17 (42.5)	
Occupational status [n (%)]			0.432
Employed	13 (31.7)	26 (72.2)	
Unemployed	28 (68.3)	11 (27.5)	
Causes of injury [n (%)]			
Road traffic accident	19 (46.3)	NA	
Fall	11 (26.8)	NA	
Sports	3 (7.3)	NA	
Violence	3 (7.3)	NA	
Disease	5 (12.2)	NA	
Level of injury [n (%)]			
Paraplegia	28 (70.7)	NA	
Tetraplegia	12 (29.3)	NA	
Severity of injury [n (%)]			
ASIA-A	18 (43.9)	NA	
ASIA-B	13 (31.7)	NA	
ASIA-C	6 (14.6)	NA	
ASIA-D	4 (9.8)	NA	
Presence of pressure sore [n (%)]			
Yes	23 (56.1)	NA	
No	18 (43.9)	NA	

SCI: Spinal cord injury; SD: Standard deviation; ASIA: American Spinal Injury Association; NA: Not applicable

**Table 2:** Health-related quality of life (HRQOL) of subjects with spinal cord injury (SCI) and healthy controls

**Variables**	**SCI group (n = 41)**			**Healthy group (n = 40)**			**P**
	**Mean ± SD**	**Minimum**	**Maximum**	**Mean ± SD**	**Minimum**	**Maximum**	
GH	42.6 ± 15.4	15	85	53.0 ± 15.3	20	90	0.001**
PF	16.9 ± 21.8	00	70	58.7 ± 20.4	35	100	0.000**
RP	46.0 ± 44.0	00	100	59.5 ± 17.7	25	100	0.101
BP	58.5 ± 21.3	00	90	73.8 ± 16.8	50	100	0.002*
VT	50.8 ± 18.3	5	9	53.7 ± 19.2	12.5	90	0.707
SF	45.1 ± 26.9	00	100	70.8 ± 28.0	12.5	90	0.000**
RE	50.6 ± 44.2	00	100	76.2 ± 27.0	32	100	0.010*
MH	64.3 ± 18.5	24	100	73.6 ± 17.2	34	100	0.029*

GH: General health; PF: Physical functioning; RP: Role-physical; BP: Bodily pain; VT: Vitality; SF: Social functioning; RE: Role-emotional; MH: Mental health; SCI: Spinal cord injury; SD: Standard deviation

* Statistically significant at the 0.05 level;

** Statistically significant at the 0.01 level

**HRQOL scores and related characteristics:** Tables 3 and 4 show that women had significantly higher scores in the PCS (P = 0.031) and MCS (P = 0.080), and in the domains of GH (P = 0.006), PF (P = 0.004), VT (P = 0.003), and SF (P = 0.001). Scores in the VT domain were significantly lower in married subjects (P = 0.030) and in those with non-formal education (P = 0.016). Unemployed subjects had significantly lower scores in the PF domain (P = 0.034). Level of injury classified as tetraplegia was significantly associated with lower scores in the PF (P < 0.001) and VT (P = 0.022) domains, and PCS (P = 0.015). Furthermore, subjects with ASIA-A had significantly lower scores in the PF (P < 0.001) and VT (P = 0.008) domains, and in PCS (P = 0.007). In addition, participants with ASIA-B had significantly higher scores in the MH domain (P = 0.037) (Tables 3 and 4).

Spearman’s correlation analysis revealed moderate negative correlation between age and the VT domain (r = -0.338, P = 0.030), and moderate positive correlation between duration of injury (28.4 ± 20.2 months) and the SF domain (r = 0.308, P = 0.050).

## Discussion

This is the first cross-sectional study, to the best of our knowledge, to explore the HRQOL of persons with SCI and its related characteristics in Nigeria. The present results show that individuals with SCI have lower scores of HRQOL in six domains of the SF-36 compared to healthy subjects. Gender, level of injury, and severity of injury were commonly related to lower scores of HRQOL.

***Sociodemographic and clinical characteristics: ***We found that the majority of the SCI subjects were men and middle-aged adults which was consistent with the findings reported in previous studies in Nigeria^3^^,^^18^^,^^19^ and other developing countries.^20^ Similar to the findings of most studies conducted in Nigeria,^18^^,^^19^^,^^21^ road traffic accidents were the most frequent cause of SCI. However, Joseph, et al. found assault (gunshots, stabbing and blunt trauma) to be the commonest cause of SCI in South Africa.^22^ Our findings that road traffic accidents is the most frequent cause of SCI may be due to over speeding, ineffective enforcement of traffic safety regulations, and dilapidated roads in the state and the country in general.^3^ Regarding the severity of injury, a greater number of the subjects suffered complete impairment (ASIA-A) which is consistent with the findings reported in the literature.^3^^,^^15^^,^^22^ Moreover, the majority of subjects had pressure sores at the time of assessment which might be attributed to the fact that many suffered complete impairment in addition to being wheelchair bounded all of which may increase the risk of developing a pressure sore.

***HRQOL scores for subjects with SCI and healthy controls: ***In the present study, subjects with SCI exhibited lower HRQOL in the domains of GH, PF, BP, RE, and MH compared to healthy controls all of which corresponds with the findings of many earlier studies carried out on the same topic.^7^^-^^9^ These findings are not surprising considering the fact that SCI has the potential to cause numerous health problems that seriously affect the physical, mental, social, and psychological aspects of the patient's life.^1^

This study found no significant differences between subjects with SCI and healthy controls in the subscales for RP and VT (energy/fatigue). Though the performance of physical roles among persons with SCI appears to be somehow 

**Table 3:** Health-related quality of life (HRQOL) of the subjects with spinal cord injury (SCI) according to related characteristics

**Variables**	**GH**	**PF**	**RP**	**BP**	**VT**	**SF**	**RE**	**MH**	**PCS**	**MCS**
Gender										
Male	39.3 ± 12.1	13.4 ± 19.6	47.8 ± 44.4	58.5 ± 21.9	46.8 ± 15.1	38.9 ± 22.2	50.7 ± 45.9	62.4 ± 17.4	31.9 ± 13.2	49.4 ± 11.6
Female	61.6 ± 19.9	37.5 ± 24.4	38.8 ± 42.4	58.7 ± 22.6	74.1 ± 19.3	81.2 ± 24.6	49.9 ± 40.8	76.0 ± 22.4	46.7 ± 14.5	71.2 ± 18.1
Marital status										
Married	41.8 ± 13.5	15.1 ± 23.0	50.8 ± 44.3	57.9 ± 20.4	45.0 ± 15.3	39.7 ± 23.3	56.2 ± 47.2	61.7 ± 17.5	32.6 ± 15.5	49.4 ± 12.8
Single	44.3 ± 19.5	20.7 ± 19.4	35.7 ± 43.9	59.8 ± 24.0	63.4 ± 18.4	56.7 ± 31.2	38.4 ± 35.6	70.1 ± 20.1	37.1 ± 11.2	59.4 ± 16.7
Education										
Formal	40.4 ± 16.9	23.0 ± 22.9	30.4 ± 40.5	56.0 ± 23.2	58.6 ± 19.8	48.8 ± 29.2	35.3 ± 36.1	63.0 ± 21.8	34.7 ± 14.1	54.2 ± 16.8
Non-formal	44.9 ± 13.8	10.5 ± 19.1	62.5 ± 42.5	61.1 ± 19.4	42.7 ± 12.5	41.2 ± 24.3	66.6 ± 47.1	65.8 ± 14.7	33.4 ± 14.8	50.9 ± 12.3
Occupational status										
Employed	45.0 ± 19.9	20.3 ± 18.4	43.4 ± 45.3	58.0 ± 21.3	62.3 ± 18.4	56,7 ± 31.2	43.5 ± 39.4	72.9 ± 18.0	37.4 ± 12.0	60.4 ± 16.8
Unemployed	41.5 ± 13.2	15.3 ± 23.4	47.3 ± 44.2	58.7 ± 20.8	45.5 ± 15.9	39.7 ± 23.3	53.8 ± 46.6	60.4 ± 17.7	32.5 ± 15.1	49.0 ± 12.3
Level of injury										
Paraplegia	44.0 ± 16.0	23.1 ± 23.2	46.2 ± 46.4	62.3 ± 21.2	54.6 ± 18.5	46.9 ± 26.8	44.8 ± 44.7	67.7 ± 18.6	37.8 ± 13.8	54.8 ± 15.2
Tetraplegia	39.1 ± 13.9	20.0 ± 4.9	45.8 ± 39.4	49.3 ± 19.4	41.6 ± 14.8	40.6 ± 27.7	64.5 ± 41.4	56.3 ± 16.3	24.9 ± 11.2	47.3 ± 12.5
Severity of injury										
ASIA-A	39.3 ± 14.4	5.2 ± 7.5	59.7 ± 37.5	62.6 ± 25.9	43.4 ± 20.2	36.1 ± 28.7	70.3 ± 42.6	64.0 ± 18.6	29.8 ± 12.1	51.0 ± 13.5
ASIA-B	46.1 ± 20.0	8.8 ± 9.3	35.7 ± 47.9	49.4 ± 19.6	54.2 ± 18.4	52.8 ± 26.5	39.1 ± 4.0	71.0 ± 19.1	29.3 ± 10.4	54.0 ± 19.4
ASIA-C	39.1 ± 3.8	47.5 ± 9.8	16.6 ± 40.8	60.8 ± 6.6	57.5 ± 2.73	37.5 ± 13.6	5.6 ± 13.6	50.0 ± 10.4	43.0 ± 9.7	48.0 ± 7.2
ASIA-D	51.2 ± 11.8	50.0 ± 33.4	62.5 ± 47.8	66.2 ± 10.8	63.7 ± 10.3	71.8 ± 6.3	66.6 ± 38.4	66.0 ± 17.4	55.0 ± 18.7	62.1 ± 6.3

GH: General health; PF: Physical functioning; RP: Role-physical; BP: Bodily pain; VT: Vitality; SF: Social functioning; RE: Role-emotional; MH: Mental health; PCS: Physical component summary; MCS: Mental component summary; ASIA: American Spinal Injury Association

Data are presented as mean ± SD.

**Table 4:** Relationship between health-related quality of life (HRQOL) of subjects with spinal cord injury (SCI) and related characteristics

**Variables**	**GH**	**PF**	**RP**	**BP**	**VT**	**SF**	**RE**	**MH**	**PCS**	**MCS**
Gender	0.006**	0.004**	0.602	0.872	0.003**	0.001**	0.900	0.201	0.031*	0.008*
Marital status	0.760	0.153	0.401	0.731	0.030*	0.221	0.530	0.672	0.171	0.483
Education	0.332	0.253	0.117	0.419	0.016*	0.586	0.149	0.318	0.562	0.810
Occupational status	0.410	0.034*	0.857	0.356	0.112	0.201	0.414	0.311	0.064	0.062
Level of injury	0.469	< 0.001**	> 0.999	0.154	0.022*	0.496	0.177	0.630	0.015*	0.147
Class of injury	0.490	< 0.001**	0.976	0.359	0.008**	0.192	0.373	0.037*	0.007**	0.254

GH: General health; PF: Physical functioning; RP: Role-physical; BP: Bodily pain; VT: Vitality; SF: Social functioning; RE: Role-emotional; MH: Mental health; PCS: Physical component summary; MCS: Mental component summary

* Statistically significant at the 0.05 level;

** Statistically significant at the 0.01 level

problematic, the lack of significant difference between these Nigerian subjects with SCI and healthy controls is surprising and may imply that the SCI subjects are able to maintain some physical role despite their illness and poor access to assistive resources. In agreement with our results, Lidal, et al.^9^ also reported non-significant difference in the RP domain between persons with SCI and the general Norwegian population. One possible explanation for the lack of significant difference between subjects with SCI and healthy controls in the VT domain might be that the mean age of these subjects (37.0 years) was lower than the mean age (41-50 years) observed in other studies.^7^^,^^9^^,^^12^ Thus, it is likely that younger age is related to greater perceived levels of energy.^8^

***HRQOL scores and related characteristics:*** Our study found that men exhibit lower scores in the GH, PF, VT, and SF domains compared to women. Additionally, similar results were obtained for the PCS and MCS subscales. In contrast with our results, other studies found no significant gender differences in any of the domains of SF-36.^9^^,^^15^^,^^23^ Our results, however, should be interpreted with caution as there were fewer women than men (14.6% vs 85.4%). The better HRQOL observed among women in this study might be because women are usually supported by their husbands and other immediate family members. Moreover, the fact that men might not be able to perform the functions of providing for their families could have affected them psychologically thereby negatively affecting their QOL.

The second determinant of HRQOL in this study was severity of injury. We found ASIA-A to be associated with lower scores of PF, VT, MH, and PCS compared to ASIA-B which is analogous to the reports of Sabour, et al.^15^ who revealed that ASIA-B fared better in comparison with ASIA-A due to some degree of preserved sensory function. Subjects with ASIA-A, however, have a complete injury with no preserved sensory and motor functions which may have contributed to lower QOL. Subjects with ASIA-D have higher scores in all the domains of HRQOL compared to the other groups. Though there were few subjects in this group, the result was not surprising since ASIA-D indicates a less severe injury. 

Patients with tetraplegia had lower scores of PF, VT, and PCS compared to paraplegic patients. An explanation for this may be that patients with more severe injury are likely to experience many health problems due to a higher level of dependency. Previous studies found that individuals with tetraplegia had poorer QOL compared to those with paraplegia, which was in agreement with the present study findings.^14^^,^^15^ Even though the paraplegic patients in our study fared better than those with tetraplegia in most domains of HRQOL, these scores were generally below average implying that the level of injury was an important determinant of QOL in this population. 

Marital status and educational level were only found to be associated with the VT domain of HRQOL. Specifically, having a non-formal education and being married were associated with lower energy scores. Even though no significant difference was observed for marital status and education level with other domains of HRQOL, these factors may be important in explaining the impact of SCI on the individual's social life. Contrary to our findings, Ebrahimzadeh, et al.^24^ and Hu, et al.^25^ found no significant difference in HRQOL across different educational levels or marital statuses. Moreover, Holicky and Charlifue reported that married individuals with SCI had less depression, greater life satisfaction and psychological well-being, and better QOL.^26^

With regard to occupation, unemployment was only found to be related to lower scores of the PF domain, which may be explained by the disability due to medical problems caused by SCI besides inadequate resources and lack of integration strategies. However, our findings are rather surprising because statistically significant difference does not exist between employed and non-employed subjects in other domains of HRQOL despite the aforementioned reasons. In another similar study, unemployment was significantly associated with lower HRQOL scores across all domains in adults living with SCI.^10^

In the current study, age was found to be negatively associated with the VT domain; this was in accordance with the findings of some previous studies, which found older age to be associated with lower scores of HRQOL.^10^^,^^13^ In contrast, other studies did not find any association between age and HRQOL of subjects with SCI.^15^^,^^27^^,^^28^ It is worthy of note that in the present study, we did not conduct a regression analysis to explore how this variable predicts the HRQOL scores considering other confounding variables. Interestingly, our study found a positive relationship between duration of injury and the SF domain, which implies that the subjects still recognized their social function despite the limited resources and lack of social integration in the community in which they lived. Other studies, however, found no relationship between time since injury and HRQOL.^24^^,^^27^

Our study is limited by the small sample size with asymmetrical distribution (skewed towards the male gender), which might not allow extrapolation of the findings. Furthermore, our subjects were recruited from public hospitals neglecting subjects in private hospitals; hence, the findings may not be generalizable. Future studies should therefore consider recruiting patients from private hospitals and other public hospitals across the country to generate data that can be generalized.

## Conclusion

This study revealed that persons with SCI in Kano, Northwestern Nigeria, experienced lower HRQOL across various domains compared to healthy controls. Gender, level of injury, and severity of injury were the major factors related to lower HRQOL scores. Other factors were marital status, educational status, age, and duration since injury. These results suggest that effective rehabilitation programs that would improve the QOL of this population are warranted.

## Notes:

**How to cite this article: **Nasidi MA, Akindele MO, Ibrahim AA, Ahmad AA, Musa A. Health-related quality of life and related characteristics of persons with spinal cord injury in Nigeria. Iran J Neurol 2019; 18(2): 50-6.
